# The Hydrophobiac Scare

**Published:** 1886-01

**Authors:** 


### THE HYDROFOBIAC SCARE.

There has been a spasm of hydrophobia scare at Newark, that has awakened the whole world within the last few weeks, owing to the running about of a furious, large, black dog, showing signs of rabis and biting several children, and at last being shot while savagely biting at a door-knob of a house on Monroe street. Every appearance of the dog was that he was suffering under an attack of rabies, but that is not yet certainly known, as no dog or no children of the several bitten, have yet had any of that terrible disease. Of course, the serious biting of so many children caused much excitement, and the end was that four children, with their mother, were shipped to Paris to be treated by M. Pasteur, who is reported to have discovered a cure for the bite of a rabid dog. They arrived in Paris on Dec. 21st, nineteen days after they were bitten, and were first operated upon that evening. We give, as briefly as possible, the manner of M. Pasteur in his practice in cases of rabies.

The matter used for inoculation is composed of veal broth, perfectly pure, exempt from any microbe, and in which has been dissolved a little of the marrow of a rabid rabbit. The broth and the marrow must be carefully prepared. The broth is made, after well-known methods, with veal which has been boiled in water. It is then put in glass receivers, of a spherical form, with long necks, which are hermetically sealed by melting the necks over a lamp. These receivers or retorts are then submitted for half an hour under pressure at a heat of 115 degrees centigrade (239 degrees Fahrenheit) in a digester somewhat similar to a Papin digester and those used by investigators in physics.

This boiling is intended to entirely destroy all germs the broth may contain. To make matters more certain the retorts are left in a stove heated to 35 degrees centigrade (95 degrees Fahrenheit). The germs develop if they have not been destroyed, and the broth becomes clouded. When it remains clear one can decant it, taking precautions for excessive cleanliness, into other little receptacles, known as Pasteur's receptacles, or into some similar receptacle.

To get the marrow one must trepan two rabbits at least each day in the laboratory. To do that the animal is stretched on a dissecting board, to which it is firmly attached by its four legs. Then it is chloroformed. You can tell if it is thoroughly asleep by its regular respiration, and having lost its consciousness it cannot resist this regular breathing by any jerking movements. The skin of the upper part of the cranium is slit longitudinally. Then, with the help of a surgical instrument known as the trepan, a small round piece is removed from the skull, great care being taken not to touch the membranes of the brain.

That done, the operator inoculates with the help of a little Pravas, or hypodermic syringe, under the first membrane, or dura mater, a little of the infected broth reserved for the purpose. He stitches together the wound and leaves the rabbit to recover. On its awakening the rabbit seems to be as little troubled as it did before the operation took place. After the inoculation of a very virulent broth rabies declares itself without fail in the rabbit at the end of six days. Two or three days later the animal dies of complete exhaustion or paralysis.

Then the marrow, care having been taken to let no foreign germ get mixed with it, is hung up in special flasks perfectly sterilized, and containing caustic potash, to thoroughly dry the air inside of them. The flasks are placed in a room carefully kept up at a heat of 20 degrees centigrade (68 degrees Fahrenheit). This exposure to a dry atmosphere and constant temperature is intended to produce a weakening of the rabic virus contained in the marrow according to the length of time that the marrow remains in the dry air. For instance, in one, two, up to ten or fifteen days, one has what M. Pasteur calls "marrow of one, two, ten or fifteen days' strength," and so on, becoming less and less virulent. When the operator wants to use it he cuts off a little round piece of about a centimetre in length, which is mixed with the broth in a quantity suitable for treating four or five persons.

The first evening each child received half a Pravas or hypodermic syringe full of broth with marrow of thirteen days' strength. An adult receives each time three-quarters of a syringe or a whole syringe full. The morning after, at eleven o'clock, each child received half a syringe of the broth and marrow of twelve days, and since the second day they have come every morning to the laboratory to receive the inoculation. Each day we take marrow which is one day younger, and therefore one day stronger than that used the day before. So the children will arrive at being inoculated at the end of about ten or eleven days with marrow of from one to two days, strength, which is certain to give a rabbit rabies by trepanning at the end of six days. It will do no harm to the children and, on the contrary, it will cure them, for the inoculations that have preceded have prepared them to receive the most virulent marrow without danger.

The first marrow of thirteen days would not kill a rabbit. That of six or seven days kills them at the end of fifteen days and does not affect the child. The treatment lasts about ten days.

There is no pain caused by this, other than the pricking of a very fine needle, to introduce the virus. There is no inflammation nor redness, unless it be on the last day, after the innoculation of the most virulent marrow ; qut there is no pain even then. During treatment the patients can go out and do what they please. It is enough that they should avoid excesses.

In most cases the treatment will not be too late, for cases are quoted where the natural incubation of rabies has not taken place for two years. The shortest time in which rabies has declared itself is forty days at least. People generally have taken the time after the bite has been received to come to Paris in order to place themselves under the care of M. Pasteur.

From all that has been said and written of M. Pasteur, it seems to be established that he has a cure for rabies, that the death of other animals is necessary to obtain it, which makes it desirable that still a better and surer mode of cure will be found ; still, this cure, if it be effectual, is one of the greatest medical discoveries of the age.

